# Hairless Expression Attenuates Apoptosis in a Mouse Model and the COS Cell Line; Involvement of p53

**DOI:** 10.1371/journal.pone.0012911

**Published:** 2010-09-23

**Authors:** Cliona O'Driscoll, Joseph P. Bressler

**Affiliations:** Division of Toxicology, Department of Environmental Health Sciences, Bloomberg School of Public Health, Johns Hopkins University, and Hugo Moser Research Institute at Kennedy Krieger, Baltimore, Maryland, United States of America; National Institutes of Health, United States of America

## Abstract

**Background:**

Neurons are more likely to die through apoptosis in the immature brain after injury whereas *adult* neurons in the mature brain die by necrosis. Several studies have suggested that this maturational change in the mechanism of cell death is regulated, in part, by thyroid hormone. We examined the involvement of the hairless (Hr) gene which has been suspected of having a role in cell cycle regulation and apoptosis in the hair follicle and is strongly regulated by the thyroid hormone in the brain.

**Methodology:**

Forced expression of Hr by transfection decreased the number of apoptotic nuclei, levels of caspase-3 activity, and cytosolic cytochrome C in COS cells exposed to staurosporine and tunicamycin. Similarly, capsase-3 activity was lower and the decrease in mitochondrial membrane potential was smaller in cultures of adult cerebellar granule neurons from wild type mice compared to Hr knockout mice induced to undergo apoptosis. *In vivo*, apoptosis as detected by positive TUNEL labeling and caspase 3 activity was lower in wild-type mice compared to Hr knockouts after exposure to trimethyltin. Hr expression lowered levels of p53, p53 mediated reporter gene activity, and lower levels of the pro-apoptotic Bcl2 family member Bax in COS cells. Finally, Hr expression did not attenuate apoptosis in mouse embryonic fibroblasts from p53 knockout mice but was effective in mouse embryonic fibroblasts from wild type mice.

**Conclusions/Significance:**

Overall, our studies demonstrate that Hr evokes an anti-apoptotic response by repressing expression of p53 and pro-apoptotic events regulated by p53.

## Introduction

A broad range of insults including hypoxia/ischemia and exposure to xenobiotics such as ethanol and anesthetics in the last trimester of pregnancy are known to have long lasting effects on cognitive and motor development [1], [2]. Different insults affect different brain regions but most cause damage by inducing cell death through apoptosis and necrosis. In the developing brain, the dominant type of neuronal cell death is apoptosis whereas neurons more frequently die through necrosis in the adult brain. Tightly controlled apoptotic mechanisms are essential for the correct pruning and formation of synaptic connections during development [3]; it is thought that these already active death pathways may be responsible for the increased vulnerability of the immature brain to insults [4]. The neurons that have failed to establish proper synapses will not survive and will undergo programmed cell death often through apoptosis. Overall, this process of neuronal and glial death assures proper matching of pre-synaptic and postsynaptic cells and, consequently, optimization in neuronal circuitry. The tendency to undergo apoptosis is enhanced because of higher levels of expression of genes that promote apoptosis such as the pro-apoptotic members of the Bcl2 family [5], [6]. In the mature brain, a shift in favor of the expression of anti-apoptotic Bcl2 family members occurs. The shift is due, in part, to thyroid hormone signaling. Hypothyroid rats display increased caspase 3 activity and increased levels of pro-apoptotic Bcl2 members and decreased Bcl2 family members in the cortex [7] cerebellum [8], and hippocampus [9].

The hairless (Hr) gene (NM_024364) is strongly regulated by thyroid hormone in the brain [10]. It is found in almost all neurons and in some white matter tracts [11]. Hr is also highly expressed in skin epithelial and the hair follicle [12]. In a series of elegant studies defining amino acid domains, Hr was shown to display co-repressor activity for several types of nuclear receptors including retinoic acid orphan receptor, thyroid hormone, and vitamin D [13], [14], [15]. In Hr knockout mice, a massive disintegration and apoptosis was observed in the bulb of the hair follicle during the first hair cycle in the mouse [12]. Interestingly, a recessive mutation in the Hr gene results in the human disease *papular atrichia*, which is characterized by complete hair loss that occurs after shedding of the first hairs [16]. The higher levels of apoptotic cells in Hr knockouts in the skin suggest that Hr is involved in apoptosis.

The objective of the study was to determine the involvement of Hr in apoptosis in the brain. Two *in vitro* models were used, which were COS cells and mouse embryonic fibroblasts genetically forced to express Hr and cerebellar granule cells from wild-type and Hr knockout mice. Additionally, apoptosis was examined in brains of wild-type and Hr knockout mice. Data will be presented demonstrating that cells expressing Hr are protected from chemically induced apoptosis. Hr appears to evoke an anti-apoptotic response by disrupting a p53 dependent pathway involving *Bcl2* family members.

## Results

### Expressing high levels of Hr attenuates apoptosis

**Figure 1:**
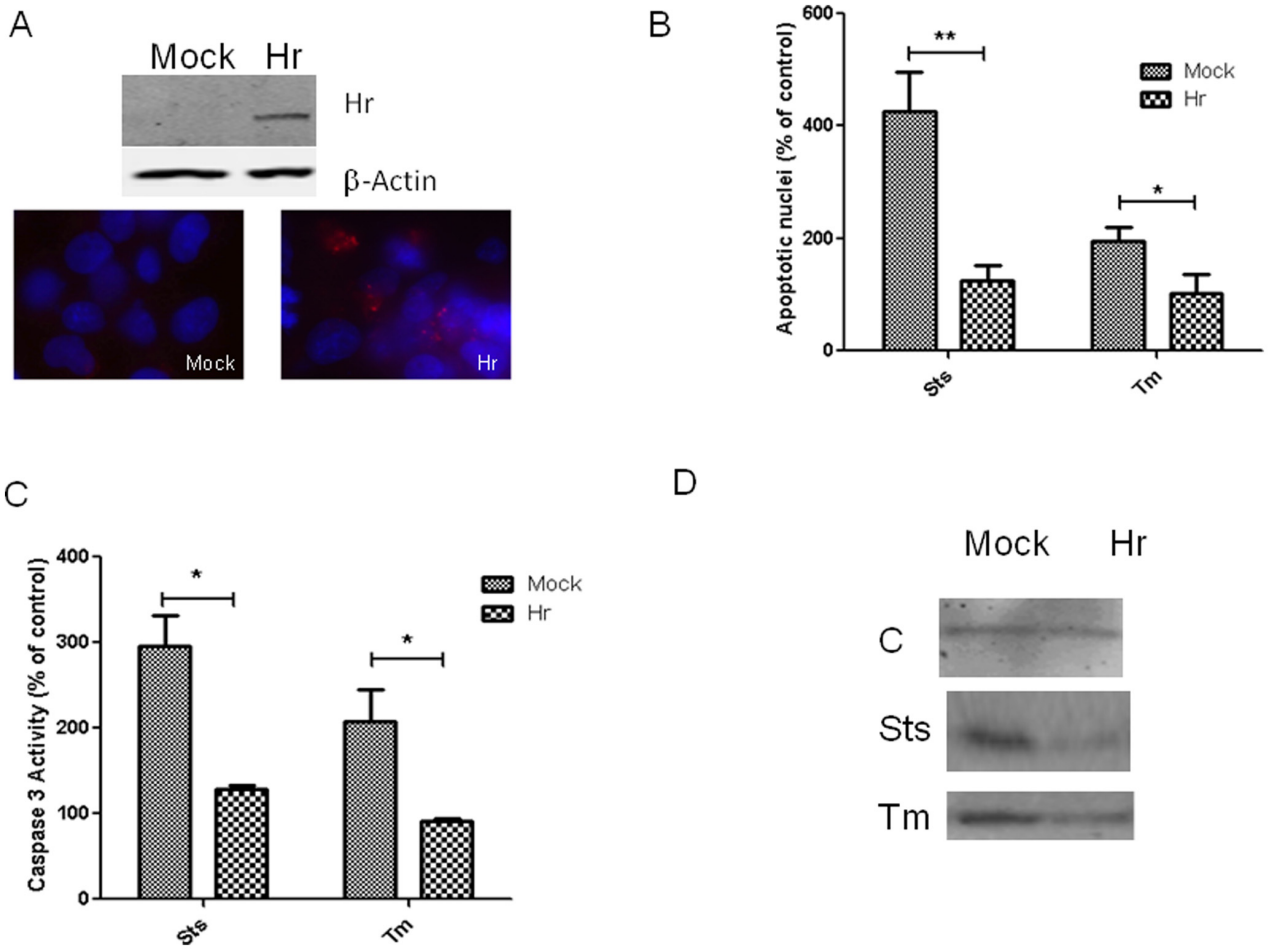
High levels of Hr expression attenuate apoptosis in COS cells. Hr was examined by Western blotting and immunocytochemistry at 48 hours after COS cells were transfected with the RK5 (mock) or RK5Hr (Hr) expression vector (A). At 24 hrs after apoptosis was induced with staurosporine (sts, 100 µM) and tunicamycin (tm 1 µg/mL), the number of cells displaying fragmented nuclei was counted after staining with DAPI (B). Caspase-3 activity was determined by measuring the release of pNA colorimetrically with the substrate Ac-DEVD-pNA (C). The percent control is computed by dividing the value from cultures treated with inducer by the value in cultures without (control) multiplied by 100. Data are expressed as means of triplicate cultures (± S.E.M.) and were repeated in three independent experiments. * indicates *p*<0.05 and ** indicates p<0.01 as determined with ANOVA and Tukey's post hoc test. To determine cytochrome C release, cytosolic fraction were subjected to Western blotting and probed with a rabbit antibody against cytochrome C and subsequent goat anti rabbit antibody with an infrared probe (D). Western blots were visualized with the Odyssey.

The effect of Hr on apoptosis was examined in COS cells by forcing expression with a transfection protocol using Hr cDNA cloned into the Rk5 expression vector or the empty vector (mock) [17]. High levels of Hr protein expression were observed in the majority of cells at 48 hrs after transfection (figure 1). When apoptosis was induced with the mitochondrial stressor staurosporine [18]and endoplasmic reticulum stressor (ER) tunicamycin [19], forced expression of Hr resulted in higher cell viability in COS cells (supplementary data figure S1). A number of parameters were examined to determine whether the changes in viability were due to effects on apoptosis. Mock transfected cultures displayed approximately four-fold more cells with fragmented nuclei than cells expressing Hr after treatment with staurosporine (figure 1). In cells treated with tunicamycin, the number of cells displaying fragmented nuclei was approximately two-fold higher in mock transfected cells compared to cells expressing Hr. Similarly, caspase-3 activity was approximately two-fold higher in mock transfected cells compared to cells expressing Hr after treatment with either chemical. Much higher levels of cytochrome C were observed in the cytoplasm of mock transfected cells treated with staurosporine and tunicamycin compared to Hr expressing cells, which indicates greater release from mitochondria. Finally, expression of Hr decreased levels of activated caspase 3 and Poly(ADP-ribose) polymerase-1 on Western blots (supplementary data figure S1).

### Cerebellar granule cells from Hr knockout are more sensitive to apoptotic inducers

**Figure 2:**
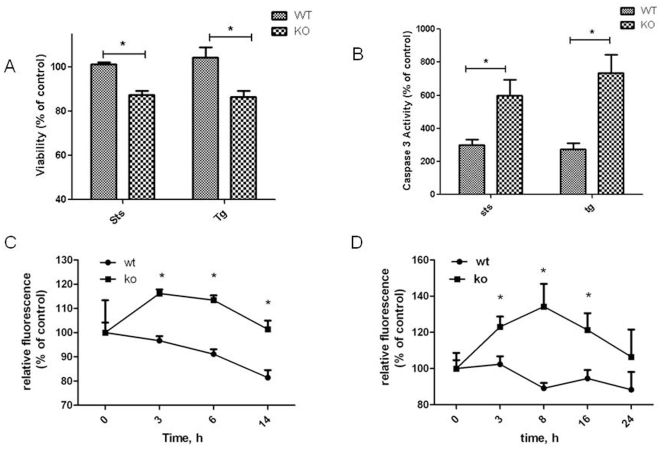
Cerebellar granule cells from Hr knockout mice display a higher sensitivity to undergo apoptosis. Cerebellar granule cell cultures were established from Hr knockout (KO) and wild-type (WT) from 7 day old mice. At 6 days after plating, cells were exposed to staurosporine (sts, 100 µM) and thapsigargin (tg, 250 nM) for 24 hrs. Viability was measured by the MTT assay in triplicates in three independent experiments (A) and caspase-3 activity was measured as described in figure 1 (B) in three independent experiments in duplicates. The outer mitochondrial membrane potential was measured in cells labeled with JC-1 fluorescent dye after treatment with staurosporine (C) and thapsigargin (D) in two different experiments in duplicates. Percent control was computed by dividing the value in the presence of the apoptotic inducer by the value in its absence and multiplying the quotient by 100. Data are expressed as means and standard error of the mean (± S.E.M.). *indicates significantly difference between knockout and wild-type at *p*<0.05 determined with ANOVA and Tukey's post hoc test.

To study the involvement of Hr in the brain, we examined apoptosis in Hr knockout mice. Cultures of cerebellar granule cells from Hr knockout mice displayed greater sensitivity to inducers of apoptosis (figure 2). Viability was significantly higher in cell cultures from wild type mice compared to knock outs after treatment with staurosporine and the ER stressor thapsigargin. The level of caspase 3 activity in cultures from knockout mice was approximately two-fold higher than levels in wild-type cultures after treatment with staurosporine and over three-fold higher after treatment with thapsigargin. Another parameter of apoptosis examined was mitochondria outer membrane potential, which decreases in unhealthy and apoptotic cells and is detected by increases in fluorescence of the JC-1 dye at an excitation of 485 nm and emission 535 nm. Decreases in potential indicate that the mitochondrial membrane has become compromised and can begin to release cytochrome C. The decrease in potential was significantly greater in cultures from Hr knockout mice treated with thapsigargin and staurosporine. The levels of fluorescence decreased at 24 hrs very likely because of cell death in the Hr knockout cultures.

### Trimethyltin (TMT) mediated apoptosis is higher in cerebellum granule layer of Hr knockout mice

**Figure 3:**
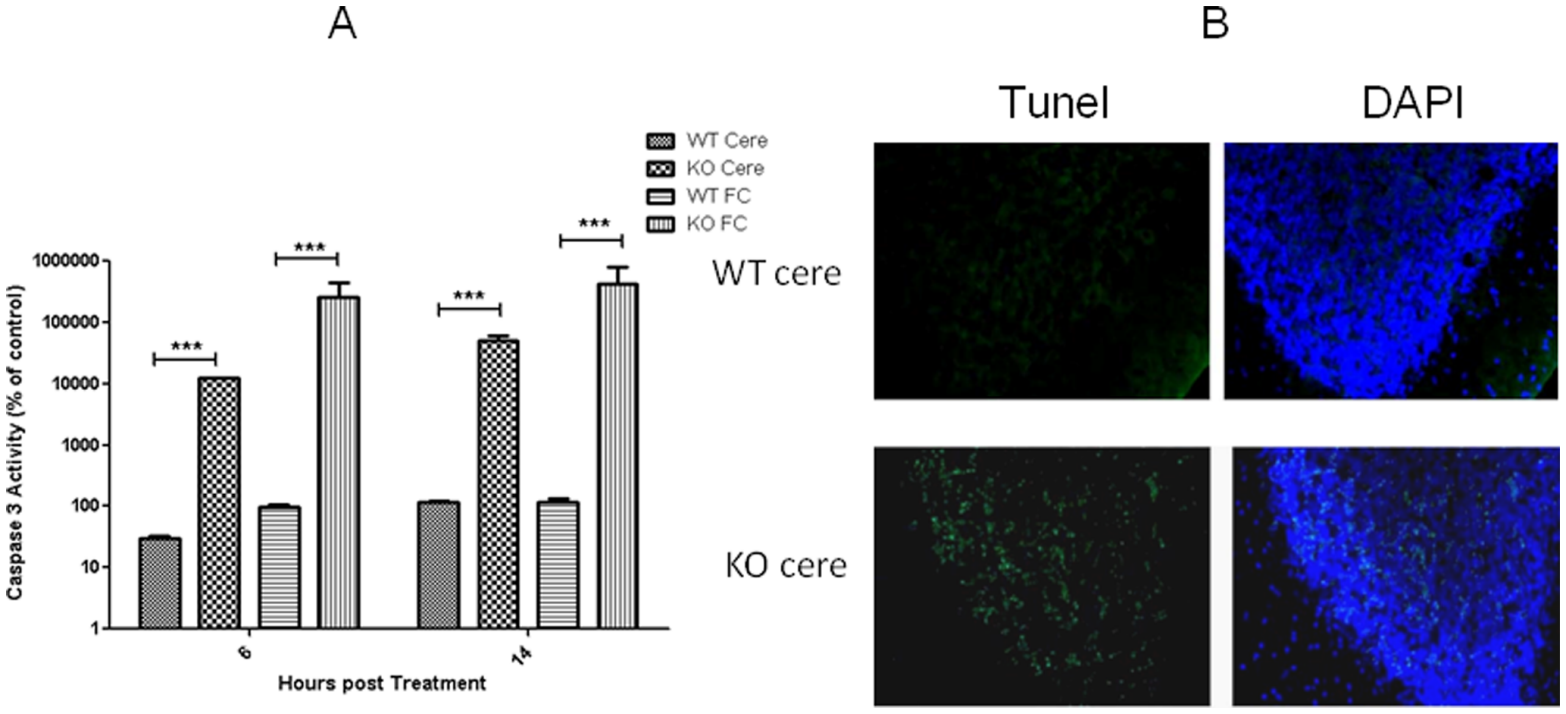
Increased sensitivity to trimethyltin in Hr knockout mice. Frontal cortex (fc) and cerebellum (cere) were isolated from brains of 25 day old knock out (KO) and wild type (WT) mice that were euthanized at 24 hrs after injection (i.p.) with 2.5 mg/kg trimethyltin. Caspase-3 activity was measured as described in figure 1 (A) in homogenates. *** p<0.001. To conduct terminal transferase-mediated biotinylated-UTO nick end-labeling (TUNEL) staining, mice were perfused with PBS/4% formaldehyde and the brains were post-fixed in the same buffer. Brains were made hyperosmotic with sucrose and sections (25 microns) were stained for TUNEL (green) and nuclei were stained with DAPI. The granule layer of the cerebellum is shown (B).

The effectiveness of Hr to attenuate apoptosis was examined in vivo by treating Hr knockout and wild-type mice with TMT, which induces apoptosis in neurons in different brain regions in rodents[20], [21]. TMT increased caspase 3 activity in homogenates prepared from cerebellum and frontal cortex in both strains but the increases were much more pronounced in the knockout homogenates at both 6 and 14 hrs. At 6 hours after treatment caspase 3 activity was 100- and 1000-fold higher in the cerebellum and frontal cortex, respectively in Hr knockout compared to wild-type mice (figure 3). We also observed many more apoptotic cells in the cerebellum as revealed by TUNEL staining in the granule layer of the cerebellum from Hr knockout mice compared to wild-type at 24 hrs after treatment with TMT.

### Hr attenuated apoptosis is p53 dependent

We next turned our attention to the mechanism in which Hr affects apoptosis. Because staurosporine and tunicamyin induce apoptosis at two different targets (mitochondria and ER, respectively), Hr could potentially be working through two different mechanisms. To determine if the early stages of the ER stress response was affected by Hr expression, levels of the protein chaperone GRP78 and the transcription factor CHOP were examined in COS cells treated with tunicamycin. Although stress was observed as indicated by increased levels of both proteins, forced expression of Hr did not reduce the amount of stress. Levels of GRP78 and CHOP were similar in Hr expressing cells and mock transfected cells (supplementary data figure S2). It is therefore unlikely that Hr affects the initiating factors involved in the stress response.

**Figure 4:**
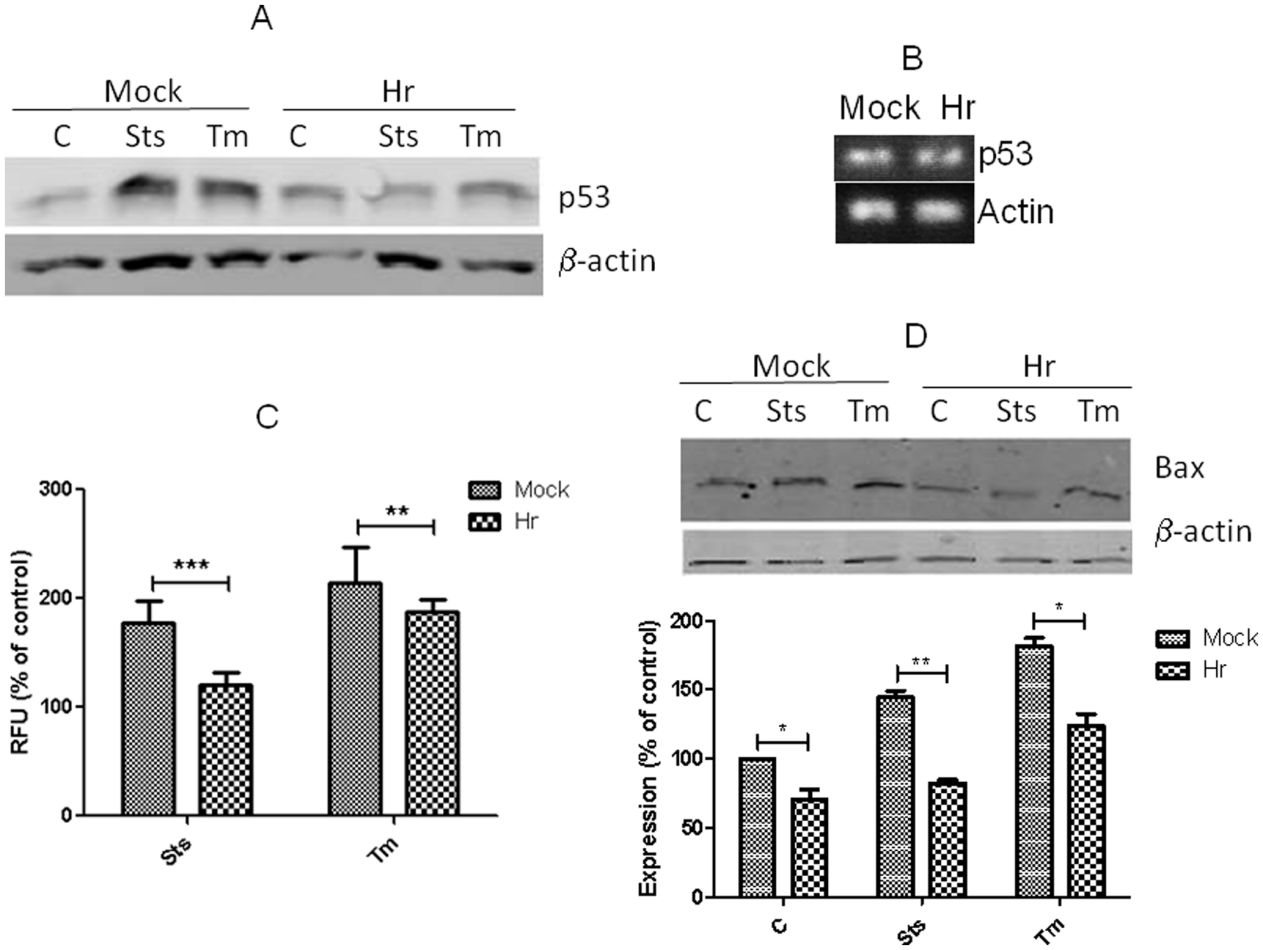
Lower levels of p53 and p53 mediated gene transcription in COS expressing high levels of Hr. At 24 hrs after COS cells were mock transfected or transfected with RK5Hr, cells were treated for 18 hours with staurosporine (sts), tunicamycn (tm), or not treated treated. Levels of p53 protein (A) and mRNA (B) were detected by Western blots and Rt-PCR, respectively. The p53 reporter assays was conducted in cells that were co-transfected with p53 luciferase reporter gene and beta-galactosidase vectors along with the RK5Hr (Hr) or RK5 vectors (mock) (C). Luciferase is expressed in relative fluorescent units (RFU) and was normalized to beta-galacotosidase activity, which was measured colorimetrically. Data are expressed as mean of n = 8 (± S.E.M.). ** and *** indicates significantly difference between knockout and wild-type at *p*<0.01 and p<0.001, respectively, which was determined with ANOVA and Tukey's post hoc test. Western blot and analysis for Bax was measured in transfected COS cells treated as described above for p53 (D). Data are expressed as mean of n = 3 (± S.E.M.). * and ** indicates significantly difference between knockout and wild-type at *p*<0.05 and p<0.01, respectively, which was determined with ANOVA and Tukey's post hoc test.

DNA damage, ER and other types of stresses induce apoptosis through a p53-dependent mechanism. Increased expression of p53 shifts the balance of gene expression in favor of pro-apoptotic family members in cells undergoing apoptosis. Also, p53 promotes apoptosis by antagonizing the anti-apoptotic effects of Bcl-xL and Bcl-2 at the outer mitochondrial membrane. As expected, the expression of p53 protein increased in mock Hr transfected COS cells treated with either tunicamycin or staurosporine (figure 4). In cells expressing Hr, the levels of p53 were much lower. No change in p53 mRNA was observed as measured by RT-PCR and real time PCR (data not shown). The lower levels of p53 protein were sufficient to affect p53 mediated transcription. In a p53 reporter gene activity assay, Hr expressing and mock transfected cells displayed increases in luciferase activity after treatment with staurosporine and tunicamycin compared to non treated cells. The increase in activity was greater in mock transfected cells (figure 4). Similarly, levels of Bax, which is regulated by p53, were also lower in cell expressing Hr (figure 4).

**Figure 5:**
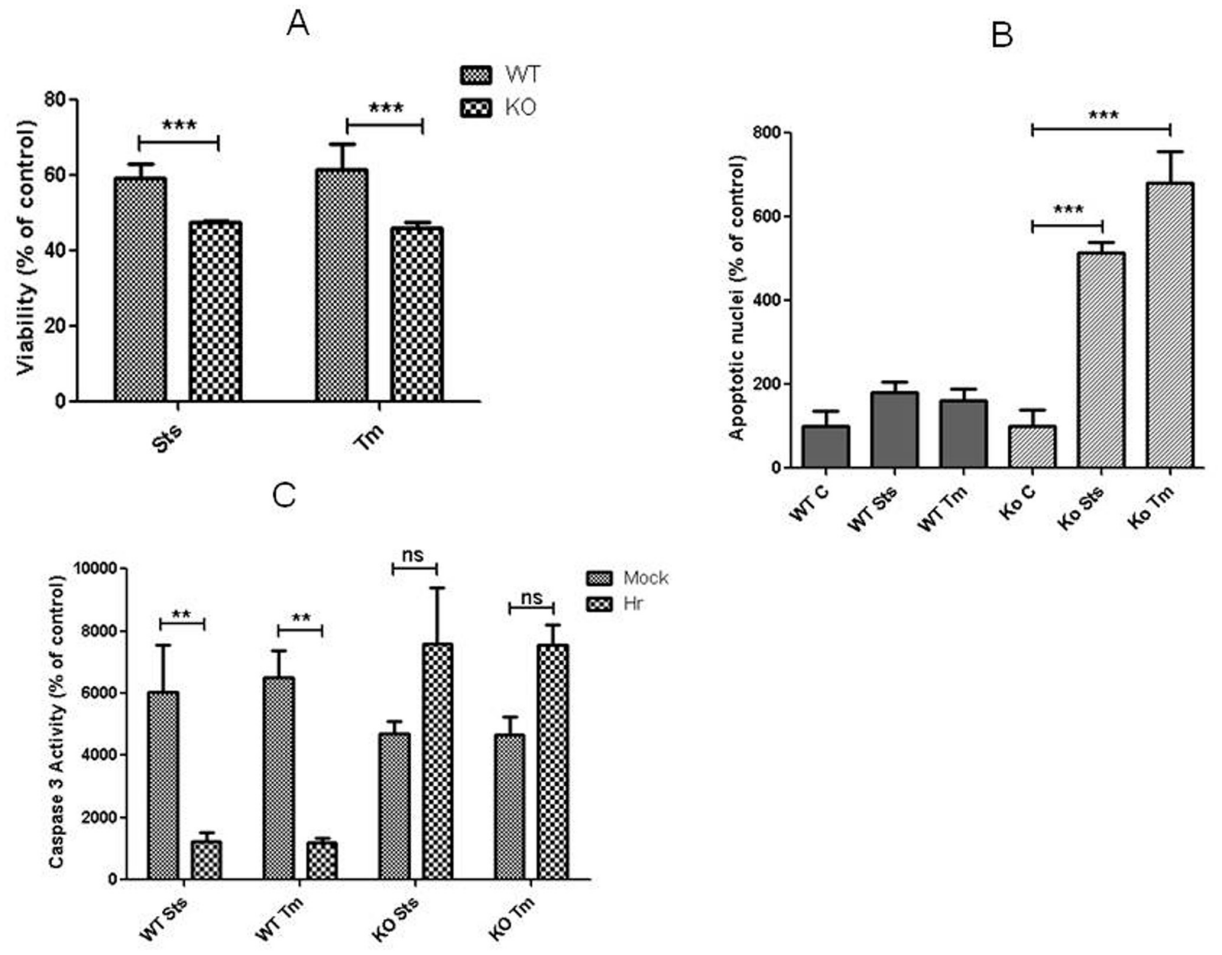
p53 is required for Hr to attenuate apoptosis. MEF from p53 wild-type (wt) and knockout (ko) mice were transfected with Rk5Hr and apoptosis was induced in cultures with staurosporine (sts) and tunicamycin (tm). Viability was measured with the MTT assay and repeated in three independent experiments in triplicate (A). The number of apoptotic nuclei was counted with DAPI staining (B). Caspase 3 activity was determined in a colorimetric assay and repeated in three independent experiments in triplicates (C). Data for viability and caspase 3 are expressed as percent control (not treated with chemicals). The percent apoptotic nuclei was determined by dividing the number of fragmented nuclei by the total number of nuclei and multiplied by 100. Data are expressed as means ± S.E.M. ** and *** indicates significantly difference between knockout and wild-type at *p*<0.01 and p<0.001, respectively, which was determined with ANOVA and Tukey's post hoc test.

To determine whether the effects of Hr are mediated through decreased expression and activity of p53, the effects of Hr on apoptosis were examined in mouse embryonic fibroblast (MEF) cell lines derived from p53 knock out and wild-type mice. Indeed, the percent increase in viability afforded by expressing Hr was significantly greater in wild type MEF compared to p53 knockout MEF after treatment with staurosporine and tunicamycin (figure 5). Neither staurosporine nor tunicamycin increased the percentage of fragmented nuclei in wild type MEF expressing Hr. In contrast, the responses of p53 knockout MEFs to staurosporine and tunicamycin were not attenuated by forced expression of Hr. Indeed, a five to six fold increase in the percent of apoptotic nuclei was observed in p53 knockout fibroblasts forced to express Hr that were treated with the drugs. Consequently, the percentage of apoptotic nuclei in MEF p53 knockouts was much higher compared to the MEF wild type after treatment with staurosporine and tunicamycin. The effect of Hr on caspase 3 activation was also affected by the p53 knockout. The percent increase in caspase 3 activity was three-fold higher in mock transfected wild-type compared to wild-type expressing Hr after treatment with staurosporine or tunicamycin. However, forced expression of Hr did not affect levels of caspase 3 activity in MEF from p53 knockout mice.

## Discussion

Two different *in vitro* models and an *in vivo* model were used to demonstrate that cells expressing Hr are more resistant to inducers of apoptosis. In the COS cell model, several criteria of apoptosis, including viability, fragmented nuclei, cytochrome C release, and caspase-3 activity were attenuated in cells expressing high levels of Hr after exposure to tunicamycin and staurosporine. Similarly, higher viability and lower caspase-3 activity were observed in cultures of cerebellar granule cells from wild-type mice compared to Hr knockout mice after treatment with apoptotic inducers. Finally, fewer apoptotic cells and lower levels of caspase-3 activity were observed in frontal cortex and cerebellum of wild-type mice after exposure to trimethytin compared to Hr knockouts. Interestingly, Hr was protective in cells treated with chemicals that induce apoptosis by interacting at different initial targets. Thapsigargin and tunicamycin induce apoptosis by stressing the ER [19]whereas staurosporine induces apoptosis by acting directly on the mitochondria [18]. Less is known on the mechanism in which trimethyltin induces apoptosis though the involvement of reactive oxygen species has been shown [22]. If Hr mediated protection was active at early stages of mitochondrial and ER stress, it would suggest that Hr exhibited multiple mechanisms. This is because stress at each organelle induces apoptosis through distinct mechanisms. In the ER stress pathway, for example, the unfolded protein response results in increased expression of protein chaperones such as GRP78 and transcription factors CHOP and ATF4 [23], [24]. The unfolded protein response was induced by tunicamycin but not attenuated by Hr in COS cells. Rather, a simpler mechanism is that Hr attenuates apoptosis at a common step in the intrinsic pathway that is induced by both ER and mitochondria stress.

Mitochondria and ER stress induce the intrinsic apoptotic pathway that involves the opening of the outer mitochondria membrane pore resulting in the release of cytochrome C and subsequent activation of caspase 3[25]. Evidence was presented indicating that Hr affects these earlier events. In COS cells, the expression of Hr resulted in less cytochrome C release from mitochondria and in cerebellar granule cells, the decrease in membrane potential was greater in cultures from cerebellar granule cells from Hr knockout mice. These changes at the outer mitochondria membrane are regulated, in part, by p53. Chemically damaged cells undergo a p53-dependent apoptotic pathway that involves upregulation of p53 transcripts such as the pro-apoptotic Bcl2 family members and p53 translocation to the mitochondria, resulting in neutralization of the anti-apoptotic members Bcl-xL and Bcl-2 [26]. In neurons, the absence or inhibition of p53 activity protects neurons *in vivo* and *in vitro* [27], [28] from acute injury and prevents cellular dysfunction induced by the mutant Huntington Disease protein product [29]. A decrease in p53 dependent transcription, and in levels of Bax, which is regulated by p53 [30], was observed in COS cells expressing Hr. Also, the anti-apoptotic effects of Hr were lessened in p53 knockout cells compared to wild-type cells. We would expect that if Hr attenuates apoptosis by blocking the p53 dependent apoptotic pathway, then the effect of Hr would be expected to be lower in the absence of p53. There are several possible mechanisms by which Hr could attenuate p53-mediated apoptosis. One possibility is that Hr decreases levels of p53 by promoting p53 degradation through a proteosome dependent pathway. p53 has previously been shown to be deacetylated through formation of a complex with the E3 ligase MDM2[31], [32], [33], and histone deacetylates (HDAC), for example HDAC1[34], resulting in proteosomal degradation of p53 [35], or p53 export from nucleus[36]. Interestingly, Hr also binds HDAC1[11], [37], [38] and possibly might also recruit a complex with p53 resulting in decreased stability through deacetylation. Another possibility is that Hr represses transcription of p53 through a mechanism involving HDAC1and a nuclear receptor. Hr has separate domains that enable it to physically interact with receptors for retinoic acid orphan, thyroid hormone, vitamin D, and possibly other receptors [14], [17], [38]. No exogenous nuclear ligand is needed because Hr is capable of repressing gene expression of unliganded Vitamin D [15], [38] and thyroid receptors [39]. This possibility is less likely because no change in p53 mRNA was observed. Also, the nuclear receptor regulating p53 is presently unknown.

In summary, the data presented indicates that Hr mediates an anti-apoptotic response involving the regulation of p53 and Bcl2 family members. The mechanism appears to involve blunting p53 mediated apoptosis by down-regulating p53 expression. Hr is developmentally regulated in the rat brain. It reaches peak expression between postnatal days 14 and 21 [10], which is similar to the peak levels of thyroid hormone serum levels in the rat [40]. We suggest that the developmental changes in Hr expression explains, in part, why cell death occurs more commonly through apoptosis in the immature rat brain whereas cell death through necrosis is more commonly observed in the adult [41], [42].

## Materials and Methods

### Tissue culture and transfections

COS cells were grown in DMEM with 10% fetal bovine serum at 37°C 5% CO2. MEF from p53 knockout mice and wild-types were a gift of Dr Stephen Jones. The calcium phosphate method [43] was used to introduce DNA into cells in a mixture containing 25 ng DNA/cm^2^. The pRK5Hr expression vector was made by inserting the XbaI–SpeI fragment of the rat hr cDNA into the XbaI site of pRK5 as previously described [17]. Cells transfected with the pRK5 plasmid under identical conditions served as the mock transfection.

### Primary culture of cerebellar granule cells

All procedures were approved by the Animal Use Committee of Johns Hopkins University and adequate measures were taken to minimize pain or discomfort to the mice. A colony of wild-type and Hr knockout mice were maintained and genotyped as previously described [44]. Cerebellar granule cells were prepared from 7-day-old mice killed by carbon dioxide asphyxiation followed by decapitation. Cells were seeded on poly-D-lysine -coated dishes at a density of 500 000 cells/cm2 and cultured in DMEM supplemented with 10% heat-inactivated fetal calf serum, 25 mm KCl, 0.5% (v/v) penicillin-streptomycin. Cytosine arabinoside (10 µM) was added to the cultures 48 h after seeding to prevent proliferation of glial cells. Cells were used for experiments at 7 days after plating and contained >95% neurons.

### Immunocytochemistry

Cells were washed and fixed in 4% paraformaldehyde for 15 min and permeabilised/blocked in 5% normal goat serum, 0.1% Triton-x 100 in PBS. Rabbit antibodies against human hairless, mouse bcl2, (both Abcam) and mouse cytochrome C (Invitrogen) were used at 1∶100 dilution. Coverslips were incubated with primary antibody overnight at 4°C, washed in PBS, and incubated with secondary antibody (goat anti-rabbit IgG labelled with rhodamine) at a 1∶200 dilution for 2 h at room temperature. The coverslips were washed three times and mounted with Prolong gold with DAPI (Invitrogen, Carlsbad, CA). The slides were viewed on the Zeiss axioplan microscope.

### Western blotting

Cells were scraped and lysates were prepared in 60 µl of RIPA (PBS, 1% Igepal, 0.5% w/v Deoxycholic acid, 0.1% SDS) buffer with protease inhibitor cocktail set I and phosphatase inhibitor cocktail set II (Calbiochem, La Jolla, CA). Pellets were sonicated to complete lysis. Protein concentration was determined by Bradford assay using Bovine Serum Albumin as standard. Cell lysates were boiled for 4 min in sample buffer containing 0.31 M Tris-HCl pH 6.8, 1% SDS, 5% glycerol, 3.6 M β-mercaptoethanol, 5 mM PMSF, and 0.05% bromophenol blue. 25 µg of protein were separated on 4–20% gradient gels (Cambrex, East, Rutherford, NJ) at 120 V. The proteins were transferred to nitrocellulose membranes at 100 V for 90 min. Nitrocellulose membranes were blocked with Odyssey blocking buffer (Licor) and incubated with appropriate primary antibody overnight. Secondary antibody (Licor) was diluted 1∶10000 in 1∶1 Odyssey buffer: PBS and 0.1% Tween and incubated with membrane for 1 h. The level of antibody binding was measured on the Licor Odyssey system and normalized to actin.

### RT-PCR

RNA was isolated using Trizol reagent (Invitrogen) according to the manufacturer's instructions. 5 µg of RNA was converted to cDNA using the superscript II kit (Invitrogen). Rt-PCR was performed with Platinium taq (Invitrogen), 1.5 mM MgCl2, 200 nM dNTPs, 200 nM each primer, 1x PCR buffer. The forward and reverse primers for p53 were CCAGCCAAAGAAGAACCAC and CTCATTCAGCTCTCGGAAC, respectively.

### p53 -luciferase reporter assay

Cells were plated into 12 well plates and transfected with 400 ng of the p53 reporter gene plasmid (#16442 and 16443 as control Addgene, Cambridge, MA) and 400 ng CMV-β-galactosidase plasmid using calcium phosphate. The p53 plasmid has 13 copies of the p53-binding consensus sequence. Cells were harvested in cell culture lysis buffer (Promega, Madison, WI) and assayed for β-galactosidase and luciferase activity using the Promega assay system. Luciferase activity was normalized to β-gal activity to correct for transfection efficiency.

### Terminal transferase-mediated biotinylated-UTO nick end-labeling (TUNEL) staining

Mice were perfused with PBS/4% formaldehyde and the brains were post-fixed in the same buffer. Brains were cryoprotected in 30% sucrose overnight and frozen. The frozen tissues were cut into 25 µm sections and stained were stained for using the Deadend Fluorimetric Tunel system (Promega) as per the manufacturers insturctions. Nuclei were counterstained and mounted with Prolong Gold with DAPI (Invitrogen).

### Mitochondrial Membrane Potential

Assay was performed as per manufacturer's instructions Mitochondrial Membrane Potential Assay Kit (Cayman Chemical). Briefly, cerebral granule cells were isolated from wildtype and hr knockout mice and plated into black 96 well plates. Cells were cultured for 7 days and treated as indicated. The cells were washed with PBS and incubated with JC1 staining solution in culture media for 15 min. The cells were then washed and analyzed on a fluorescent plate reader. JC1 monomers form in apoptotic and unhealthy cells and can measured at an excitation of 485 nm and emission 535 nm.

### Apoptotic Nuclei Counts

Cells were plated onto Coverslips and treated as indicated in the figure legends. Cells were fixed in 4% PFA and washed in PBS. Coverslips were then mounted using Prolong with DAPI. Cells in which the nucleus contained clearly condensed chromatin or cells exhibiting fragmented nuclei were determined to be apoptotic. Apoptotic data are reported as percentage apoptosis, obtained by determining the numbers of apoptotic cells *versus* the total number of cells. A minimum of 3 counts (minimum of 100–200 cells/count) were included for each treatment.

### Cytochrome C release

Cells were plated into 100 mm dishes and treated as indicated in the figure legend. Mitochondrial and cytoplasmic fractions were isolated using the Mitochondria/Cytosol Fractionation Kit # K256-100 (Biovision). Briefly, following treatment cells were harvested and lysed using the Cytosol Extraction Buffer Mix with DTT and Protease Inhibitors, cells were incubated on ice for 10 min prior to being homogenized in a dounce homogenizer. The homogenate was centrifuged at 3000 RPM for 10 mins and the supernatant was retained. The supernatant was then spun at 13,000 RPM for 30 min to collect the mitochondrial pellet. The protein concentration was determined by Bradford assay. 10 µg protein was run out per sample on a 12% SDS-PAGE gel, the gel was probed with mouse antibody against cytochrome C (1∶1000, Biolegend).

## Supporting Information

Figure S1 (0.43 MB TIF)

Figure S2 (0.70 MB TIF)
