# Supplementary figures and images for: Hairless Expression Attenuates Apoptosis in a Mouse Model and the COS Cell Line; Involvement of p53
Source: PLoS One. 2010 Sep 23;5(9):e12911. doi: 10.1371/journal.pone.0012911 (PMC2944824; doi:10.1371/journal.pone.0012911)

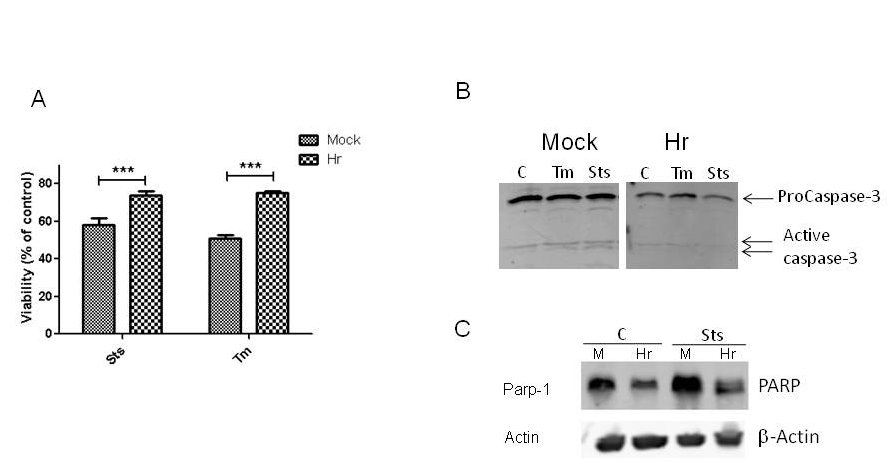

Supplement: Figure S1 — (0.43 MB TIF) [file pone.0012911.s001.tif]

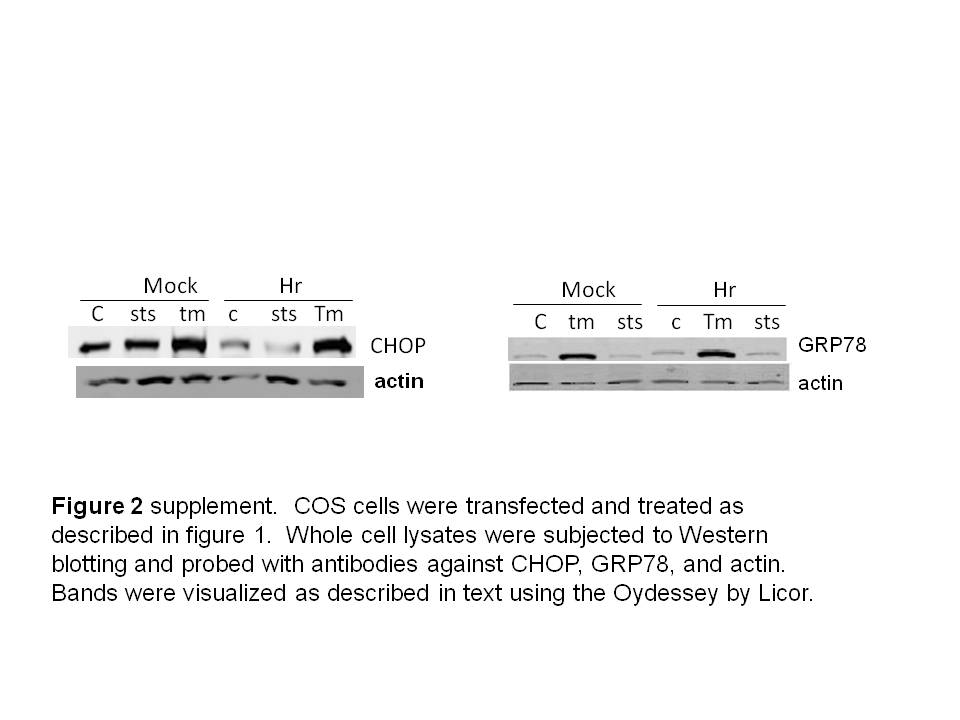

Supplement: Figure S2 — (0.70 MB TIF) [file pone.0012911.s002.tif]
